# Shaped by asymmetrical interdependence: a qualitative case study of the external influences on international non-governmental organizations’ implementation of equity principles in HIV/AIDS work

**DOI:** 10.1186/s12939-014-0086-2

**Published:** 2014-10-08

**Authors:** Elizabeth Dyke, Nancy Edwards, Ian McDowell, Richard Muga, Stephen Brown

**Affiliations:** Independent Health Consultant, Gatineau, Canada; School of Nursing, University of Ottawa, Ottawa, Canada; Department of Epidemiology & Community Medicine, Faculty of Medicine, University of Ottawa, Ottawa, Canada; Great Lakes University of Kisumu, Kisumu, Kenya; School of Political Studies, University of Ottawa, Ottawa, Canada

**Keywords:** Equity, HIV/AIDS, Non-governmental organizations, Vulnerable populations, Case study

## Abstract

**Introduction:**

Addressing inequities is a key role for international non-governmental organizations (INGOs) working in health and development. Yet, putting equity principles into practice can prove challenging. In-depth empirical research examining what influences INGOs’ implementation of equity principles is limited. This study examined the influences on one INGO’s implementation of equity principles in its HIV/AIDS programs.

**Methods:**

This research employed a case study with nested components (an INGO operating in Kenya, with offices in North America). We used multiple data collection methods, including document reviews, interviews (with staff, partners and clients of the INGO in Kenya), and participant observation (with Kenyan INGO staff). Participant observation was conducted with 10 people over three months. Forty-one interviews were completed, and 127 documents analyzed. Data analysis followed Auerbach and Silverstein’s analytic process (2003), with qualitative coding conducted in multiple stages, using descriptive matrices, visual displays and networks (Miles and Huberman, 1994).

**Results:**

There was a gap between the INGO’s intent to implement equity principles and actual practice due to multiple influences from various players, including donors and country governments. The INGO was reliant on donor funding and needed permission from the Kenyan government to work in-country. Major influences included donor agendas and funding, donor country policies, and Southern country government priorities and legislation. The INGO privileged particular vulnerable populations (based on its reputation, its history, and the priorities of the Kenyan government and the donors). To balance its equity commitment with the influences from other players, the INGO aligned with the system as well as pushed back incrementally on the donors and the Kenyan government to influence these organizations’ equity agendas. By moving its equity agenda forward incrementally and using its reputational advantage, the INGO avoided potential negative repercussions that might result from pushing too fast or working outside the system.

**Conclusions:**

The INGO aligned the implementation of equity principles in its HIV/AIDS initiatives by working within a system characterized by asymmetrical interdependence. Influences from the donors and Kenyan government contributed to an implementation gap between what the INGO intended to accomplish in implementing equity principles in HIV/AIDS work and actual practice.

## Introduction

Addressing inequities is a key role for international non-governmental organizations (INGOs) working in health and development. Yet, putting equity principles into practice can prove challenging, and there is a lack of in-depth empirical research on what influences INGOs’ implementation of equity principles in their work. The present study helps to fill this gap by using a case study to examine INGOs’ implementation of equity principles in their HIV/AIDS initiatives. This study helps to illustrate the nature of the implementation gap between the intent of INGOs to ensure equity in their work and actual practice, and examines the various influences that affect the implementation of INGOs’ equity principles.

Inequities in health are defined as inequalities that are unjust, unfair, and unacceptable, yet avoidable [1]. Inequities in health status, access to care and health outcomes are growing within and between countries [2] and are therefore squarely on the global development agenda. Concerns over growing inequities were a major stimulus for the World Health Organization’s Commission on Social Determinants of Health, established in 2005 [2,3], and the importance of addressing inequities was reaffirmed in the 2011 Rio Political Declaration on Social Determinants of Health [4]. Both called for collaborative action by multiple players from government to civil society. In the Rio Declaration, achieving health equity was described as a “shared responsibility [that] requires the engagement of all sectors of government, of all segments of society, and of all members of the international community” [4], p. 1.

### Defining International Non-Governmental Organizations

No single definition exists for non-governmental organizations (NGOs), which complicates comparing data and literature on them^a^ [5]. However, common definitional elements of an NGO include a formal, independent structure that is not-for-profit, voluntary, and has goals for the betterment of society [6-8]. International NGOs working in health and development typically have wide geographic coverage using a structured system of offices that are located in both Northern and Southern^b^ countries [6,9]. These organizations typically have broad development aims that include improving the overall health of people in Southern countries by providing health services, addressing determinants of health (e.g. water and sanitation), and/or providing humanitarian aid [6,7]. Examples of INGOs active in sub-Saharan Africa include CARE, World Vision, Oxfam, AMREF, Médecins Sans Frontières, Save the Children, and Plan [6,7].

### Equity, INGOs and HIV/AIDS

INGOs are important global health players. Many cite equity as a goal in their visions, missions and strategic directions, and also work with the most vulnerable in communities [10-13]. The increase in the number of INGOs attests to their critical role and the reliance of donors on them for implementing projects in Southern countries. The number of active INGOs worldwide increased from 9,396 in 1981 to 23,071 in 2011 [14]. NGO funding from donor countries for population activities (including HIV/AIDS work) increased from $816 million in 1999 to $4.6 billion in 2009 [15], p. 19. The United Nations Population Fund reported that in sub-Saharan Africa in 2009, 44% of donor country aid for population activities (including HIV/AIDS work) was channeled via NGOs, with the remaining funding through bilateral (34%) and multilateral (23%) channels [15], p. 19. Donor countries’ reliance on INGOs for aid distribution illustrates the critical role that INGOs play in the health and development of Southern countries.

This study uses HIV/AIDS initiatives as the exemplar given the magnitude of the problem, the efforts of the international community to address the epidemic, and the inequities in HIV/AIDS. Addressing HIV/AIDS is one of the Millennium Development Goals [16], reflecting its immense human toll. Worldwide, approximately 34 million people were HIV positive in 2011 [17], p. 8, of whom 23.5 million were in sub-Saharan Africa, where 1.8 million new infections were reported in 2011 [17], p. 14. In 2009, almost US$16 billion were spent in response to the HIV/AIDS epidemic globally; in Southern countries, including those in sub-Saharan Africa, 88% of resources for HIV/AIDS came from “international funding”, particularly from bilateral (high-income country government) donors [18], p. 145.

Countries in sub-Saharan Africa typically have a generalized HIV/AIDS epidemic, which means that HIV/AIDS is not limited to certain subgroups, and is transmitted in the general population [19]. However, within this generalized epidemic, certain groups are more vulnerable and at higher risk for HIV/AIDS (including women, men who have sex with men, injecting drug users, and sex workers), due to inequities in access to care as well as upstream determinants of health that increase their social and political vulnerability [17,18].

### Role of INGOs in enhancing health equity

NGOs may pursue their equity mandates through service delivery and/or advocacy [6,7]. Service delivery can help to fill short-term needs for vulnerable groups, often filling gaps that are the result of cuts to government services [7]. Advocacy can be an important means for NGOs to influence the equity-related agendas of other players such as governments and donors. Advocacy contributes to broader and sustainable reductions in inequities by modifying upstream determinants of heath [7,20] and may counteract NGOs’ perceived closeness to governments and donors – closeness which may limit NGOs’ ability to advocate [7,20]. However, some NGOs report that Northern countries and donors drive the equity agenda, limiting local input [21].

Despite INGOs’ efforts in equity, including in HIV/AIDS work, significant inequities remain. While INGOs are not the only group responsible for addressing inequities, an implementation gap exists between the intent of INGOs to ensure equity in their HIV/AIDS work and actual practice. This implementation gap is identified in general terms in the NGO literature, in particular referencing issues of continuing or deepening poverty in sub-Saharan Africa, despite the increased number and influence of NGOs working in health and development [22]. Lewis identified that INGOs have multiple internal accountabilities (to their staff and their boards) and external accountabilities (to donors, country governments, and beneficiaries) [5], one potential contributor to this implementation gap. Najam also made reference to these multiple accountabilities: donors provide much of the resources for NGOs’ work; country governments provide the legal space in which NGOs operate; communities are the beneficiaries of NGOs’ services and have expectations regarding these services, while NGOs have their own strategic directions on which to implement and report [23]. However, while formal mechanisms are in place for accountability to donors (via reporting requirements) and country governments (through registration as an organization and permission to continue to conduct work in the country), there are minimal formal mechanisms for accountability to the actual communities where NGOs work [5,23], particularly since INGOs are not typically membership-based organizations [9].

Some authors postulate that reliance on donor funding and close relationships with governments can lead an NGO to focus on the needs of the donors and country government at the expense of beneficiaries, resulting in concerns about the lack of independence of the NGO if it is funded by donors and working closely with governments [7]. If an NGO is spending its time delivering services, is dependent on donors for funding and is working closely with donors and governments, its advocacy agenda on behalf of the community (if applicable) will be limited [24], especially if the NGO fears repercussions such as losing donor funding [7,25]. However, if NGOs act as a substitute for government in service delivery, this may perpetuate neo-liberalism [26], making it difficult to shift the paradigm (including NGO advocacy for government responsibility in providing essential services in education and health) [27]. Both scenarios can challenge an NGO’s autonomy in addressing its own agenda (including equity), since the NGO has to ensure that it aligns with the donors’ and the government’s agendas [7,25]. NGOs are not elected bodies, and beneficiaries have little power to hold them accountable [25]. Hence, questions arise about the mechanisms of accountability NGOs have in working with the most vulnerable, and whether NGOs can legitimately represent beneficiaries (though advocacy efforts) or address the needs of these groups (through service delivery) [7,25].

### Multiple influences on INGOs’ implementation of equity principles

Limited empirical research has been conducted to understand what influences an INGO’s implementation of equity principles. Studies that have been conducted have used a variety of methods, including document reviews, participant observation, interviews, and surveys. Previous research has focused on gender mainstreaming [28-31] and poverty [21] rather than a broader view of equity. These studies show that NGOs encounter multiple challenges moving from strategy to action in implementing equity principles.

One challenge identified was the limited involvement from beneficiaries. Despite the rhetoric of the importance of community participation in poverty programming, those living in poverty had limited involvement in selecting priorities for interventions [21].

Sub-saharan African countries’ cultural norms can challenge NGOs’ move from stated commitment to action on equity, and these cultural norms can also influence a community’s commitment to equity [28,29]. For instance, one study reported that governments in four sub-Saharan Africa countries (Zambia, Rwanda, Uganda, and Gambia) tended to resist work on gender equality, due to beliefs that it was a donor-driven concept and not suitable for their cultural reality [30].

Changing cultural attitudes and behaviours takes time. Although gender training for staff was commonly reported, it was viewed as an insufficient means to shift longstanding gender-related attitudes and behaviours. Thus, gender training did not consistently yield desired results [29-31]. While some NGOs may have formally committed to the idea of gender mainstreaming, NGO interventions at the community level tended to address immediate needs but not the underlying determinants of health (e.g. addressing broader issues of gender including women’s rights and issues of power, violence and abuse) [29,31].

Monitoring equity is also an important activity. NGOs’ equity-focused data collection (if any) tended to focus on monitoring access to interventions (e.g. comparing the number of women and men participating in programs) [28,31], rather than examining (and addressing) equity of outcomes or shifts in underlying determinants of health that influence equity.

#### Methodological strengths and limitations of previous studies

Strengths of some prior studies include the use of multiple data collection methods (e.g. interviews, document analysis, and/or participant observation) [28,29], in some cases with multiple NGOs [21,28-31] and/or across several countries [21,30]. These approaches allow for important comparisons and the triangulation of different perspectives. However, methodologies typically focused on collecting data from internal NGO staff, and did not typically include data collection from multiple players (e.g. from government, donors, partner NGOs, community) to help understand the external and internal influences on implementing equity. None included the perspectives of staff working for the same INGO in Northern and Southern countries, limiting comparisons on how staff view equity influences within INGOs. The focus of prior research has also been on gender and poverty, rather than a more broad view of equity.

The current study extends previous research by examining the Kenyan and Northern offices of one INGO, as well as multiple players that have the potential to influence equity implementation, principally donors and the Kenyan government. The present research also examines dimensions of equity that go beyond gender and poverty. This study addressed the following questions: 1) What is the nature of the implementation gap between the intent of an INGO to ensure equity in its HIV/AIDS work and its actual practice? 2) What characterizes multi-level influences that affect an INGO’s implementation of equity principles in its HIV/AIDS work? 3) How do multi-level influences affect an INGO’s implementation of equity principles in its HIV/AIDS work?

## Methods

### Kenya as the setting for the case study

Kenya was the primary case study setting as it has a high prevalence of HIV/AIDS (6.3%) [18] and documented inequalities in the distribution of HIV prevalence, access to care, and HIV knowledge and behaviours (including between women and men, among different geographic settings, and for certain vulnerable populations such as men who have sex with men, sex workers, and injecting drug users [32-34]). It also has a large number of INGOs (with 367 responding to a survey by the Kenyan NGOs Co-ordination Board in 2009) [35], p. 25.

### Overview of the case study

A case study with nested components [36] was conducted on a major INGO working in development and health in Kenya, addressing “how…the subunit connect[s] with other subunits and the whole” [36], p. 152. The nested subunits included the Kenyan INGO and the Northern offices of the INGO in Canada and the United States. Data were collected in 2010 using multiple methods, offering rich, in-depth data, and allowing for data triangulation [37]. During a three-month period in Kenya, the first author (ED) conducted participant observations with INGO staff; interviews with these staff, its clients (interviews conducted by a Kenyan research assistant) and external partners (including staff from donor organizations, Kenyan government, and other NGOs); and document analysis. Following this, interviews and document collection took place in the Northern offices of the INGO in Canada and the United States (with one interview conducted by telephone).

In keeping with the lead author’s philosophical stance, the case study approach of Stake was primarily followed, including acknowledging ED’s background [37,38]. Where appropriate, some aspects of Yin’s approach were incorporated, in particular conducting a literature search prior to the fieldwork, as well as developing propositions and general interview questions prior to entering the field [39].

### Selection of the INGO for the case study

To choose an INGO in Kenya, eligibility criteria included that the organization: had an office in Kenya, worked in many different countries (with offices in Northern and Southern countries, including Northern offices in Canada, the U.S., or the United Kingdom), had been in operation for a long period of time, had significant resources (from multiple donors) and influence [6,9], and had declared equity principles.

Four eligible INGOs initially agreed to in-person meetings to discuss taking part in the research. The final selection of the case INGO was based on availability and willingness to take part, and on the opportunity for ED to participate as a volunteer within the organization during the data collection period.

The case INGO was formed over 30 years ago, and has multiple offices in sub-Saharan Africa and in Northern countries. The staff and programs related to HIV/AIDS formed the boundaries [38] of this case. To maintain anonymity of the case INGO, it will be referred to as “the INGO”.

### Eligibility and recruitment of participants

Employees at the Kenya office were approached for the participant observation component if they worked for the INGO in an overarching capacity (e.g. senior management, evaluation) or specifically on HIV/AIDS projects. ED conducted the participant observation as an “active member” [40], p. 50. She took part in day-to-day activities and conducted work assigned to her, including writing and editing documents related to HIV/AIDS. ED met staff through introductions and attending meetings. In addition, the manager in charge of the HIV/AIDS area emailed a communiqué to the heads of all of the HIV/AIDS projects to outline the research, stating that ED would be speaking with them or their staff about participating in the project if they were willing to take part. ED then met individually with staff from all of the HIV/AIDS projects, as well as other relevant staff, to discuss the research and provide them with participant observation information forms for their review. ED asked each of them to review the forms, invited them to pose any questions they might have, and then, if they were willing to take part, to sign the consent form. ED wrote field notes [38] about daily work in the INGO. An observational protocol was developed to assist with note-taking in the field [37], including descriptive notes and reflections [38]. The field notes captured information about the overall context of the INGO and its partners, as well as observations regarding how equity is thought of and operationalized on a day-to-day basis.

After approximately six weeks of participant observation, ED started to conduct interviews. Table 1 outlines eligibility criteria for the interviewees.

**Table 1:** **Eligibility criteria by interview group**

**Interviewee type**	**Eligibility criteria**
Internal Interviews	
-INGO staff in Kenya and INGO staff in Canada and the U.S.	-Had been employed for more than three months at the INGO (to ensure that they had sufficient knowledge of the issues)
	-Was currently working (i.e. not on leave)
	-Was currently involved with the development, delivery, or evaluation of HIV/AIDS initiatives, or involved in the INGO’s work across projects including HIV/AIDS (e.g. senior management, communication, evaluation)
External interviews (partners of the INGO)	-Had a relationship with the INGO Kenya office in their HIV/AIDS work through their organization, if this relationship had been in place for over six months
Client interviews	-Was a current or past client of one of INGO’s HIV/AIDS programs
	-Was 18 years of age or older, and capable of providing informed consent
	-Had been a client for more than six months (to adequately answer the questions)

ED sought to interview staff from each of the HIV/AIDS projects underway at the INGO, and both senior managers and junior project staff, seeking maximum variation [41]. External interviews were held in Kenya with people familiar with (but not employed by) the INGO. This included Kenyan government staff, donors, and staff of other NGOs who were partners with the case INGO. ED recruited external staff based on suggestions from RM and from key contacts in the INGO, to achieve maximum variation [41]. ED interviewed representatives from the two major Kenyan government structures that focused on HIV/AIDS at the district and national levels (the National AIDS Control Council and the National AIDS and STI Control Programme). She also interviewed representatives from two large Northern government donors that provided funding to the case INGO’s HIV/AIDS projects. Partner NGO representatives were purposively selected from two Kenyan-based NGOs that worked in partnership with the case INGO on HIV/AIDS.

The INGO had seven projects underway on HIV/AIDS. Two of these projects were not eligible for client interviews: one project was just starting and the other was in the final reporting phase. Two contrasting projects that differed in terms of services offered and geographic coverage were selected [41]. One project involved the distribution of anti-retroviral therapy (ART), embedded within an integrated INGO health facility, while the other focused on capacity building and granting funding for civil society organizations working in HIV/AIDS. Clients for both projects were recruited through the INGO program. An INGO staff person approached clients and read a prepared script to see if the client had any interest in speaking to ED’s research assistant in more detail about the research. If they did, the research assistant then approached them to discuss the research and their potential interest in being interviewed. To help ensure clients did not feel coerced, the organizational consent form signed by a senior Kenya staff person committed to “ensuring that there are no negative consequences for clients who participate in the research, regardless of what clients reported”.

For the ART project, clients were selected in discussion with the research assistant, INGO project staff, and ED to ensure that a mix of clients from different components of the project were interviewed. For the capacity building project, clients were selected based on discussions with the INGO project staff and ED, and depending on scheduled field visit follow-ups that the research assistant and ED were attending. For both projects, a mix of males and females was also sought.

Interviews were also conducted with staff from the Canadian and the American INGO offices following data collection in Kenya. Given the small number of staff in these offices, all staff who fit the eligibility criteria outlined in Table 1 were interviewed.

It was determined that a sufficient number of interviews had been conducted once data saturation was reached, or when all those fitting the eligibility criteria were interviewed (e.g. Northern offices).

### Data collection

Data collection took place between February and July 2010.

Ten staff who worked for the INGO agreed to be observed, and these observations took place between March and May 2010. Two staff were senior managers overseeing HIV/AIDS work and one worked on evaluating projects, including HIV/AIDS projects. The remaining staff worked for one of the INGO’s HIV/AIDS projects: one staff member each for three of the projects, and four from the largest HIV/AIDS project. Nine of these participants were located in the Nairobi office, and one was located in Eastern Kenya.

A total of 41 interviews were conducted. Interviews were held with INGO staff in Kenya, the U.S. and Canada, external partners, and clients (see Table 2).

**Table 2:** **Summary of interviews conducted**

**Interviewee type**	**Number of interviews conducted**	**Sex**	**Interviewee’s location**	**Staff level**
**TOTAL INTERVIEWS = 41**				
Internal interviews (INGO Staff in Kenya)	16	8 males	14 Nairobi	6 senior staff
		8 females	2 Western province	5 managers
				5 non-managerial
External interviews (Partners of the INGO)	8	5 males	5 Nairobi	2 senior staff
		3 females	2 Western province	4 managers
			1 Nyanza province	2 non-managerial
Client interviews	10	5 males	6 Nairobi	N/A
		5 females	4 Western province	
INGO interviews in Canada and the U.S.	7	4 males	1 Canada	1 senior staff
		3 females	6 U.S.	4 managers
				2 non-managerial
**Subtotals**	**41 interviews**	**22 males**	**34 Kenya (25 Nairobi; 8 Western province; 1 Nyanza province)**	**9 senior staff**
				**13 managers**
		**19 females**		**9 non-managers**
			**7 international**	**10 clients (not applicable)**

In Kenya, 16 interviews were conducted with internal INGO staff between April and May 2010. The interviews lasted on average 64 minutes (ranging from 32 to 120 minutes). In addition to internal INGO interviews, eight external interviews were also conducted in Kenya during this same time period — two with donors, four with government officials and two with partner Southern NGOs. The external interviews lasted 30 to 75 minutes, averaging 47 minutes. Over this period, ten interviews were conducted with clients of the INGO’s HIV/AIDS projects- – six with the its anti-retroviral therapy (ART) project and four with the its capacity-building and grant-making project. The client interviews lasted 23 minutes on average (ranging from 17 to 34 minutes). In June and July 2010, one interview was conducted in Canada and six in the United States with staff from the INGO office headquarters in Canada and the U.S. These Northern office interviews averaged 47 minutes (ranging from 37 to 60 minutes). All interviews were done in person, save one U.S. interview that was done by telephone. The Canadian staff person worked on proposal writing and technical support for projects in Kenya and other Southern countries, including HIV/AIDS projects. The U.S. interviewees included senior staff, as well as project, finance, and communication staff who worked with Southern country offices, including the Kenyan office, on various projects including HIV/AIDS.

For each set of interviews (i.e., internal Kenya staff, external staff, Northern staff, clients), ED developed an open-ended interview guide. The questions for the INGO and external interviewees asked how they and their organization viewed equity, and what challenges and influences they experienced in operationalizing equity in HIV/AIDS initiatives. Client interviews focused on understanding the clients’ involvement and perceptions of the INGO’s HIV/AIDS programming, including any advice they had previously provided to the INGO on the HIV/AIDS project with which they were involved, what the INGO had done to help the client be involved in the program, and what the INGO could do to encourage more clients to access the program. The draft interview guides were pilot tested with three individuals prior to ED’s arrival in Kenya. These individuals all had experience working with other NGOs, HIV/AIDS, and vulnerable populations. Their experiences included work with Aboriginal populations in Canada, pastoral communities in Tanzania, and women in Southeast Asia. The changes suggested were minor, and were incorporated into the final versions.

Following each interview, field notes were written using a contact summary form to capture reflections and key points on the interviews, and suggestions for follow-up (e.g. other people to interview, documents to access, additional probes for future interviews). ED conducted all interviews except those with clients. A Kenyan research assistant was hired and trained to conduct the individual client interviews; the research assistant also completed the interview contact form. Prior to the start of the client interviews, the research assistant translated all of the relevant documents, including information form, consent form, and interview guide into Kiswahili. RM and one of the INGO staff then verified the translations. Clients were offered the opportunity to be interviewed in Kiswahili, but most chose to speak in English, using occasional phrases in Kiswahili that were then translated into English by the research assistant during the transcription process. Clients were provided with a small travel honorarium, corresponding to the INGO’s typical travel honorarium amount.

Documents were analyzed to examine what was formally written about the INGO’s implementation of equity principles in its HIV/AIDS work and influences, and to substantiate and supplement data from participant observation and interviews [39]. Documents of interest included those relevant to the case (e.g. relevant to HIV/AIDS and/or equity at the donor, government, INGO, or community level), including project materials for HIV/AIDS initiatives underway and conducted in the past (e.g. project files), evaluations of projects, annual reports, minutes of pertinent meetings (e.g. senior management team minutes), requests for proposals from donors, responses to these requests for proposals from the INGO, and government and donor strategies that might influence the INGO. A document summary form was developed to make notations on the relevance, significance and key points from the documents. Approximately 120 eligible documents were gathered in Kenya, and seven each in the U.S. and Canada. Eighty-eight of the Kenyan documents were INGO documents and included senior management minutes, equity-related guidelines for the organization, human resource information, strategic plans, governance information, project meeting materials, responses to requests for proposals, and project reports (including evaluations, needs assessments, monthly and annual reports, implementation plans, brochures, and training information). Twenty-two Kenyan government documents included strategic plans, governance information, operating plans and frameworks, and evaluation, data and indicator documents. Ten were donor documents, including strategic plans and requests for proposals. Documents provided by the Canadian office included proposals, newsletters and annual reports. The U.S. office provided an annual report, program information, a newspaper article, a request for proposal, and governance policies. The majority of these documents covered the time period from 2006 to 2010, while a few were from as early as 2003.

### Ethics

This project followed the guidelines of the Canadian *Tri-Council Policy Statement on Ethical Conduct for Research Involving Humans* [42]. Prior to recruitment and data collection, the research was assessed and approved by the Health Sciences and Science Research Ethics Board at the University of Ottawa [43], the ethics committees at the Great Lakes University in Kisumu, and the Kenya Medical Research Institute. Participants were provided with an information sheet, informed that participation was voluntary, and invited to take part. Information and consent forms clearly outlined the benefits and risks of taking part in the research. The interviews were digitally recorded if the interviewee consented.

#### Data access and storage

ED had access to the audio recordings and transcriptions. The research assistant and transcriptionists had access to perform their work, and each signed confidentiality forms. During the fieldwork in Kenya, ED stored the data in a secure location (locked cabinet) at her residence. Once ED returned to Canada, she stored all data in a locked cabinet in her home office. She stored electronic materials on her laptop with password protection. Upon completion, verification, and member checking of the transcripts, ED erased all tape recordings. Once data analysis was complete, ED stored all raw and analyzed data on a password-protected storage device, and deleted the data (including NVivo files, transcripts, back-up files) from her computer. Upon publication of the research, all paper files and the password protected storage device will be stored in a locked cabinet at the office of NE at the University of Ottawa, to be held for five years. As per ethics requirements, at the end of five years, all confidential paper documents (e.g. consent forms) will be shredded and data on the storage device will be deleted.

### Analysis

ED led the analysis using Auerbach and Silverstein’s process (2003) [44]. Throughout all stages of the analysis process, repeating ideas, categories, and themes were discussed and reviewed with NE and IM. ED immersed herself in the data and developed thick descriptions of various components needed for the case studies of the INGO’s seven HIV/AIDS programs, including details of funding, objectives, start and end date, target groups, location, activities, budget and staffing. While fieldwork was still underway, early descriptive visual displays as per Miles and Huberman [41] were developed to represent the multiple players influencing the INGO’s implementation of its equity principles in these programs.

NVivo 9 [45] was used to assist with the initial coding of all interview data. First-level coding entailed identifying text relevant to the research questions. These segments of text were then grouped into “repeated ideas” [44], p. 36–37; no pre-structured coding framework was used at this initial stage. Participant observation and document data were reviewed, and relevant text coded to new or to existing codes (from the interviews) as applicable. “Structural coding” was then completed for data from each interview question [46], p. 84. Descriptive matrices were developed [41] using full quotes to compare and contrast challenges and facilitators of equity by different respondents (e.g. donors, Kenyan government, clients, Kenyan INGO staff, Northern INGO staff). For second-level coding, repeating ideas were reviewed and grouped into categories based on their commonalities. At this stage, various drafts of “networks” [38,41], p. 93 were drawn to explore emerging relationships between common categories as a basis for an emergent conceptual framework. During third-level interpretive analysis, overall themes arising from the case study were developed. ED wrote the integrated storyline based on the interrelated nature of these themes, going back and forth between the raw data and the themes arising to ensure coherence and to further develop and refine the themes.

### Data verification and preliminary dissemination

To verify the raw data, all interviewees in Kenya (apart from the client interviews) and North America were offered the opportunity to member check their interviews and provide any feedback from this review. One year following data collection, ED verified the preliminary analysis through an in-person presentation and discussion of results with Kenyan-based INGO staff. In addition, a written summary of the preliminary findings was emailed to all staff who had taken part in the research in Kenya, the U.S. and Canada, and staff had the opportunity to review and comment on the written version of the preliminary findings.

## Results

### Explicit identification of equity by donors and the Kenyan government

Equity was explicitly identified as a principle in many of the donors’ and Kenyan government’s recent documents, and many interviewees noted that these players’ explicit identification of equity helped the INGO in its implementation of equity principles in HIV/AIDS. For example, in both donor documents and interviews with donors, gender equity was identified as a consistent domain of focus:

*“Equity comes mostly under the ambit of gender at [donor organization]” (#606, donor).*

However, donors’ focus on equity differed *–* for some, equity was more central to their work, including a human rights focus. Generally, European donors were viewed as being more “*equity conscious*” than U.S. donors *(#400, government staff),* and certain countries within Europe, such as Sweden, were viewed as having a particularly strong equity orientation.

Kenyan government documents cited equity as a key principle for health in general and HIV/AIDS in particular [33,47,48]. Other overarching government documents, including Kenya’s 2010 Constitution (which was in draft form at the time of data collection) had a major equity focus.

### Interdependence of players working on HIV/AIDS in Kenya

The INGO worked interdependently with donors and the Kenyan government. This interdependence was reflected through their reliance on each other in working towards a common goal of responding to and addressing the HIV/AIDS epidemic in Kenya. The major role of the donors was the provision of funding, while the Kenyan government coordinated the overall country response to HIV/AIDS, and the INGO implemented programs at the community level. Each needed the others to move towards their goal.

### How the INGO implemented equity principles in its HIV/AIDS work

Many interviewees noted that the INGO implemented its equity principles in its HIV/AIDS work by focusing its programs on vulnerable groups where the INGO was viewed as having a positive and long-standing reputation. These included geographically remote populations *(“We’ve done fairly well on [working in the furthest regions] historically*” (#205, Northern INGO staff)) and people living in poverty *(“[The INGO] has always focused on…the poor population*” (#2, internal staff)). Addressing equity by targeting these groups was viewed as ingrained in the INGO’s work. Interviewees also identified that the INGO implemented its equity principles in its HIV/AIDS work by focusing on gender. Staff frequently equated equity with gender equity: *“In my perception, equity is more seen as male and female” (#85, internal staff).* Much of the INGO’s approach to addressing gender inequities involved empowering communities to strengthen health systems. The ultimate goal of empowerment, according to the INGO staff interviewed, was to enable community members to advocate their community’s health needs on their own behalf and increase their involvement in making health decisions:

*“[The INGO’s] main mission in our strategy in Africa is to empower… communities, the least empowered of them [to achieve] an equitable health status, which entails… teaching, training in health promotion, disease prevention, and providing the health systems and the human resources for health and everything that’s involved in health systems, the medications, the devices, equitable access – all the areas that fall under health systems strengthening, in an equitable manner in those communities. So that’s also giving them a voice… and giving them training about demanding the equity and achieving the equity” (#204, Northern INGO staff).*

As this quote illustrates, empowerment entailed more than service provision. For example, the INGO’s empowerment approach built capacity of community organizations and community members to ensure they had the necessary knowledge and expertise to provide services and advocate on behalf of the community, and linked the community with government structures to ensure community participation in planning fora and on formal committees. The INGO also worked closely with cultural custodians (e.g. through chief’s *barazas*^c^ and councils) on issues of culture, to increase their understanding of gender issues and the benefits of empowering women by changing cultural practices that perpetuate inequities:

*“Talking about [gender] in barazas and other forums so that they can advocate for the rights of women” (#59, internal staff).*

Training these community leaders in gender equity was viewed by a number of INGO staff as critical to the INGO’s work on gender empowerment, as this was aimed at shifting structural conditions, including cultural norms and practices.

### Shaped by interdependence: external influences on the INGO’s equity implementation in HIV/AIDS

The interdependencies of the INGO, government, and donors shaped the INGO’s implementation of equity principles. The INGO aligned its implementation of equity principles in its HIV/AIDS work to the agendas of the donors and Kenyan government. The INGO implemented initiatives and focused on certain populations based on this alignment.

#### Aligning with priorities of donors and Kenyan government

Many INGO staff stated that working with the Kenyan government, including adhering to its priorities and policies, had been key to the success of the INGO in implementing equity in its HIV/AIDS work.

*“You’ll not hear the Ministry of Health saying that [the INGO] is implementing a project that doesn’t adhere to its rules and regulations or policies or guidelines… You need the government to be fully behind an initiative, and if they are not behind it, you find that it’s very difficult” (#16, internal staff).*

The INGO’s community empowerment approach aligned well with the Kenyan government’s Community Strategy, which had as a major focus to empower communities in health related issues:

*“The intention [of the Community Strategy] is to build the capacity of communities to assess, analyze, plan, implement and manage health and health related development issues… communities will thereby be empowered to demand their rights to seek accountability from the formal [health] system for the efficiency and effectiveness of health and other services… The goal of reducing health inequities can only be achieved effectively by involving the population in decisions and in the mobilization and allocation of resources, and thereby promoting community ownership and control in the context in which they live their lives”* [49]*, p. 2, 4.*

Government staff confirmed the Kenyan government’s commitment to the Community Strategy:

*“I think our interest is not to simply have community members as recipients of services and goods, our interest is to empower them, so that they make decisions, they help us in decision-making and sustainability of those programs…. So an area of interest to us is empowerment, so that empowerment of communities to take part in decision-making and promote sustainability of programming, even in the absence of support that is external” (#603, government staff).*

Hence, the INGO’s community empowerment approach aligned with the Kenyan government’s priorities.

The INGO’s work in gender empowerment in its HIV/AIDS programs aligned with donor agendas, as gender equity was an area of priority for donors and an explicit funding condition of some donors. A few interviewees identified donors as the impetus for the INGO’s focus on gender equity:

*“In Kenya, we weren’t looking at an equity lens until donors came in and said ‘Women have rights too and they need to have equal chances’…Now most programs look at gender issues” (#50, internal staff).*

The INGO’s focus on gender equity also aligned with the Kenyan government’s equity work, including the government’s collection of HIV/AIDS data by sex. The Kenyan government required that the INGO (and all NGOs and civil society organizations working in HIV/AIDS) complete a quarterly report on activities, and all data had to be reported for males and females separately (e.g. number of people trained, number receiving food and nutrition support). Internal interviewees also identified disaggregation of data by sex as the primary way that equity was measured by the INGO: “*In our reporting, we make sure that our data is disaggregated by gender” (#33, internal staff)*. Civil society organization clients funded by the INGO were also required to report data for males and females as part of their civil society organizations’ activities:

*“[The INGO] wants to see the management of organizations. Is it dominated by men alone, or is it inclusive? Are ladies represented?” (#508, client)*.

The formal collection of data by sex illustrated how the donors’ and Kenyan government’s priority of gender equity filtered to the INGO, and down into the community.

Hence, two of the major ways that the INGO implemented equity in its HIV/AIDS work were through community empowerment and a gender equity focus, aligning with priority areas for the donors and the Kenyan government.

#### Aligning with donor funding: “He who pays the piper calls the tune”

Donors primarily influenced the INGO’s implementation of equity in its HIV/AIDS work through funding requirements: *“He who pays the piper calls the tune” (#400 – government staff and #124 – internal staff).* Since the INGO relied heavily on donor funding, this influence was strong:

*“At the end of the day, [the donor] is only concerned with what’s in the proposal [in terms of whether or not equity is a focus]” (#205, Northern INGO staff).*

Specific requirements for funding proposals meant that INGO staff often felt that they lacked the flexibility to insert their own ideas and principles, which could limit the opportunity to realize fully the INGO’s equity principles in its projects. For example, some interviewees observed that when the donor’s request for a proposal for an HIV/AIDS project was not oriented towards equity, if INGO staff included an equity focus, the INGO might not be awarded the funding.

*“Because the other thing that has to be kept in the forefront, is that the RFA [request for applications] has very specific outputs. And it is only those outputs that you are allowed to respond to, in response to that RFA. And so, for example, if there isn’t anything in there about equity, and then you put specific programmatic things around, you’re going to add that, you are not responsive to the RFA. And you are automatically denied” (#201, Northern INGO staff).*

*“Why struggle to suggest something in the proposal when, first of all, the instructions for doing the proposal are very rigid. You are told exactly then how to write each paragraph and what to say, what targets to meet… But indeed sometimes they say suggest other innovative ideas. And if equity is one of them, it might fit. Or if a disadvantaged region of the country or communities or groups are, then that’s when you might have scope” (#3, internal staff).*

However, some INGO staff felt that many donors facilitated the INGO’s implementation of equity in HIV/AIDS, since equity was often a focus of donor funding.

*“I’d say that donors facilitate [equity] more than anything else. I mean, after all, their funds come from them, but also… the donors are coming from countries where equity probably is more advanced. To them, it’s also of key interest that we demonstrate that whatever we’re implementing [in HIV/AIDS] is addressing equity, that it is basically making the lives of communities better. So, donors actually facilitate [equity]” (#1, internal staff).*

As one interviewee noted, equity as a whole may be a donor-driven concept: *“everyone is moving on equity because this is all Western driven, the labels are Western, they are part of donor conditionalities”(#124, internal staff)*.

Donors had particular political ideologies and values that defined what they wanted to focus on, and fund, in terms of HIV/AIDS programs. Donors’ requests for proposals were based on the priorities and agenda of the Northern country first and foremost.

*“As a development agency [from Northern country], we have our global policy that guides the overall development. This comes from external research, evidence, and our experience in development work in countries” (#607, donor).*

Many interviewees noted that certain donors, in particular those from European countries, were more likely to include an equity focus in their requests for proposals for HIV/AIDS projects.

*“The proposals [for funding of HIV/AIDS initiatives] originating from Europe tend to embrace equity in this broader sense as in pro-poor, explicitly, as compared to… the American [proposals]” (#3, internal staff).*

By contrast, the U.S. was identified as one donor that, in the past, had deliberately excluded certain projects that facilitated equity. The HIV/AIDS funding under the Bush Jr. administration had criteria that some interviewees felt negatively affected the implementation of equity principles.

*“We’ve had a long period where [for U.S.’s PEPFAR funding] family planning has been off the agenda…which doesn’t help in any way, because the well-off people – they are always getting access to family planning, the ones that are well-educated and know what to do. The poor people with less education, with poorer access, they are then the ones that are marginalized in this case, right? So, some donors, because of their policy, are limiting the ability to have a proper equity, or fully, fully focus on equity… and some are very supportive” (#2, internal staff).*

This same senior staff person noted that donors’ priorities, and the resulting requests for proposals, changed depending on the donor country’s current political climate:

*“USA, especially PEPFAR… moves with the wind depending on who is in power and the government system” (#2, internal staff).*

The U.S. was viewed as having increased its focus on equity under the Obama administration, and following the development of the Global Health Initiative (from PEPFAR).

*“Increasingly, even the American proposals you see that they want that you target the most vulnerable in society” (#3, internal staff).*

This shift was identified in interviews, and verified in participant observation and in the review of proposals for new HIV/AIDS projects, in which the U.S. recently added equity as one of its focus areas.

Because donors’ requests for proposals reflected their Northern country’s political ideologies and values, a few interviewees, both external and internal, acknowledged that donors had the potential to override what Kenya had identified as priorities by prescribing projects in requests for proposals:

*“So you might find that if a [donor] doesn’t really care about gender or maybe with MARPs [most-at risk-populations] or anything else, it’s very difficult for us…Those are decisions made already in Washington. It’s very difficult for us to say, ‘No, we have a gap addressing girls in school” [or] ‘you need some equity element to that’. They’ll tell you ‘No, but it’s not in our plans so we won’t do it’” (#400, government staff).*

Even with formal mechanisms to encourage alignment of donor funding with Kenyan government priorities in HIV/AIDS (including the Paris Declaration [50], the Action Framework for HIV/AIDS [51], and the Kenyan Health Sector Wide Approach – Code of Conduct [52]), equity elements of HIV/AIDS programs were particularly susceptible to donor influence.

*“There are some [donors] that look at what they need to get out of [funding a project on HIV/AIDS in Kenya] and they give their resources to that. The [government] doesn’t control resources… The best we can do as [government agency] is to provide the best guidance, and say, ‘Okay, fine. This is the minimum package, this is the policy, this is what you need to do, this is how you get consent, this is how it happens’…. We can try and tell them, ‘Okay fine, I think you need to work in this particular region’, but we are not always successful to tell them that we need to work in this particular region, because the mechanisms that these partners and the donors have… we may not have total control for that” (#400, government staff).*

Hence, donors had the ability to direct local implementation decisions that influenced equity. This included decisions on whether vulnerable groups, including those in certain remote geographic areas, were a focus for HIV/AIDS interventions.

Interviewees also noted that donors influenced the INGO’s implementation of equity principles in its HIV/AIDS work via the duration of funding. Project funding was typically short-term (between three and five years), which was not viewed as conducive to fostering equity:

*“Donors’ cycles are insufficient for addressing equity issues… Donors just want to provide services” (#41, internal staff).*

Providing short-term services may address immediate needs, but it is inadequate to shift structural determinants, including cultural changes. These short-term agendas were often a result of the Northern country’s political cycle of four or five years, but, as noted by interviewees, making a difference in terms of equity, including empowering communities, can take many years *(#201, Northern government staff).*

*“If you would have a program that would last, let’s say six years, then it would be more meaningful in terms of [equity] outcomes, ‘cause we’re really based on outputs, ‘cause it’s really – it’s changing perceptions and building that capacity that we have been building” (#85, internal staff).*

Hence, a disconnect existed between the donors’ needs (to focus on short-term outputs to report to constituents) and development needs (which would include long-term equity outcomes).

#### Aligning with Kenyan government’s legislation and donor countries’ policies

The Kenyan government and donors also shaped the INGO’s implementation of its equity principles in terms of where the INGO *did not* focus. Senior managers noted that the INGO did not advocate legislative changes to address human rights for most-at-risk populations^d^, including men who have sex with men and sex workers.

The INGO had an excellent reputation, built on its history of work in the communities and its positive relationships with donors and the Kenyan government. The INGO wanted to maintain this reputation and therefore avoided advocating on issues that would be perceived as highly controversial or out of step with Kenyan legislation.

*“So we push for the whole area of equity and address vulnerability and marginalization as much as we can, especially in the areas that are not political controversial” (#2, internal staff).*

Senior management staff of the Kenyan INGO said in interviews and in participant observation that if these parameters were not respected, the INGO risked a number of consequences that would have a direct and deleterious impact on their work in the country. These included being banned from Kenya if it were to implement its equity principles through advocating certain controversial legal changes, such as legislation to legalize homosexuality:

*“If you want, as an organization, to go out and advocate for legalization of homosexuality, then you would be in trouble… Well, if we did, it would have serious consequences for us; or it could have… I mean, in the most extreme cases, we would be deregistered as an NGO, right, and not be allowed in the country. That would be on the extreme, but you know, it could be more control [over] what we’re doing, lobbying donors for not giving us money, us in our ability to do policy influence in our other areas…and, [the INGO] wouldn’t go that way because it has never been the way we have been working, trying to be confrontational” (#2, internal staff).*

For this reason, the INGO worked within the system in ways that avoided potentially serious repercussions. For example, in Kenya, homosexuality, sex work, and drug use are illegal. Advocating the legalization of homosexuality or sex work, or providing harm reduction services to injecting drug users, were viewed as contentious given legal frameworks and socio-cultural norms in Kenya. As one partner NGO interviewee noted, in order to maintain positive relationships with the Kenyan government, many NGOs avoided advocating changes to legislation in these highly contentious areas.

*“[Some NGOs] are looking at their relationship with the government and they realize that issues of homosexuality are criminalized in this country and so they realize that to maintain a good relationship with the government, they don’t want to be talking about [legalization]. Sex work again is criminalized and therefore organizations are cautious to look at [legalization]” (#602, partner NGO).*

The case INGO was one of these NGOs that worked within the Kenyan legal framework to maintain these relationships:

“*So we try and find ways of working within the present legal framework [of the country], whether we agree to it or not” (#2, internal staff).*

From an equity perspective, working within the legal framework limited the programming and advocacy approaches that the INGO could take with most-at-risk populations, including men who have sex with men and sex workers. As one interviewee explained in speaking about one of Kenya’s neighbouring countries:

*“It means we can’t make statements on the issue [of legalizing homosexuality]. And we did not make statements about Uganda—the penalties [death penalty] for being gay, the legislation that was pending recently. And… that’s because the countries in which we work, it’s illegal. So, we try to be very neutral as an organization and I regret that” (#204, Northern INGO staff).*

The INGO refrained from advocating controversial issues, opting instead to remain “neutral” (i.e., silent) in order to continue working closely with Southern country governments. The quote above from a Northern INGO staff member also highlights the challenge when legal frameworks in Southern countries contradict equity values of Northern donor countries. As a Kenyan INGO staff member noted:

*“You will have some of the offices that… would try and push issues that are difficult for us to address, again homosexuality. Some of the national [Northern] offices will say, “You must fight for [legalizing homosexuality]”. [Interviewer: And how does that play out?] “We will say what we can do [in Kenya] and what we can’t do, so they have not pushed us on areas where we just can’t implement it. So they [Northern offices] might have a strong desire, but so far they have listened to us and then agreed to disagree, when we have said, ‘We can’t move on that’” (#2, internal staff).*

This illustrates the tension between the Northern INGO and Kenyan INGO staff who had different perceptions about how equity could be implemented and the risks involved. While the Northern INGO staff may have sought to advocate on these issues, the Kenyan INGO continued to priorize aligning with the Kenyan government to maintain positive relationships, avoid negative repercussions, and continue its work.

Contradictory directives within Kenya existed as well. While the Kenyan government identified the importance of working with most-at-risk populations in the Kenyan National AIDS Strategic Plan III (KNASP), there was incongruence between these statements and the legislation Kenya has in place. This challenge was directly acknowledged in the KNASP:

*“A series of difficult legal issues arise from attempts to programme more directly for the MARPs (sex workers, IDUs, MSM), and to take these programmes to scale. Sex work, homosexuality and drug use are all illegal in Kenya. Programmes have been working with all these groups for many years, but under constraints. There is a need to come up with policies that will facilitate scaling up access to services by the different groups clustered under the term MARPs”* [48].

A few interviewees indicated that the Kenyan government and the case INGO focused on public health advocacy, as outlined in the KNASP, including reaching vulnerable groups with health services. Another tactic would have been to advocate equity through an overall human rights approach, including advocating for changes in legal frameworks for most-at-risk populations. However, a few interviewees noted that the Kenyan government focused on a public health approach, which included health care access, rather than a human rights approach that would involve addressing human rights issues for most-at-risk populations by changing legislation. As one interviewee explained:

*“There is the public health [approach] which is what the KNASP looks at. Then there is the human rights approach, which is what some organizations want to look at… The KNASP is really talking about prevention and care for these populations [which is a public health approach], but we have some organizations also that are going into… issues of legislation like having them not criminalized [which is a human rights approach]” (#602, partner NGO).*

Hence, providing health services to various groups, including most-at-risk populations, would fall under what was deemed acceptable under the KNASP and permissible within the legislative framework in Kenya. However, the INGO did not move beyond this to focus on a more human rights approach given potential repercussions.

A donor country’s policies could also influence the INGO in its implementation of equity principles. An instance was cited where the donor country’s policies had the potential to negatively influence the INGO’s implementation of its equity principles in its HIV/AIDS work. In order to be eligible to receive U.S. funding, the INGO agreed to the U.S.’s policy forbidding its funding recipients to advocate the legalization of sex work. By agreeing to this policy, the INGO could not advocate any change to the legal framework in Kenya while holding this U.S. funding. One of the Northern INGO staff identified this as a limitation to the INGO’s right to advocate on issues that might influence equity:

*“This type of thing [signing this agreement] can be a challenge though as what if we wanted to advocate for a change in law in the country we are working in? There was some contention since we had to have this policy if we wanted the grant, but we might want to support the legalization of prostitution at some point, so this encroaches on freedom of speech” (#200, Northern INGO staff).*

Thus, the INGO’s reliance on donor funding could also place a limit on the potential advocacy levers that the INGO could use in implementing its equity principles in its HIV/AIDS work.

Hence, legislation and policies from other players in the interdependent system within which the INGO worked influenced the INGO’s advocacy work or the potential areas for the INGO’s advocacy. In particular, the INGO was constrained in its human rights efforts.

## Discussion

### Asymmetrical interdependence

The present research clearly shows the significant role that INGOs play in equity, and the importance of understanding the multiple players and levels that influence an INGO’s implementation of equity principles in HIV/AIDS. However, a gap exists between the intent of INGOs’ implementation of equity principles and actual practice due to multiple influences from various players, including donors and country governments.

Discussions in international relations refer to “asymmetrical interdependence” between nations [53], p. 37. This concept aptly describes the relations among INGOs, government, and donors. The donors, the Kenyan government, and the INGO were interdependent as they worked on HIV/AIDS in Kenya. The donors provided the necessary funding, the Kenyan government coordinated Kenyan’s overall response to HIV/AIDS, and the INGO was a key implementer of programs in specific communities. This interdependence was asymmetrical, however, as the INGO did not have equal power to the Kenyan government or to the donors in implementing its equity principles. This asymmetry arose, first, because the INGO relied on donors for its funding. Previous literature shows that not only do NGOs rely on donor funding, they compete for donor funding [54,55], competition likely augmented by the large (and increasing) number of NGOs working in Kenya [35,56]. Second, the INGO depended on the Kenyan government’s approval to operate in Kenya, and the Kenyan government oversaw the overall strategy and coordination of HIV/AIDS in Kenya. Critical to asymmetrical interdependence is the notion of power: “potential power accrues to the less dependent actor in a relationship” [53], p. 37. Much of the discussion about power in the NGO literature focuses on the reliance of NGOs on donor funding, and the resulting “overt” influence [57], p. 6 that the donor has on the NGO [58]. Dimensions of power include control over financial resources as well as political authority, leading to the ability to assert a level of dominance over other players, including the ability to set the agenda [59-63]. This reflects the findings from the present research, namely the financial power from the donors and political power of government. Many interviewees noted how donors *“still drive how organizations operate” (#300, Northern INGO staff)*, and potential repercussions from the government were a consideration in implementation of equity principles. Many authors speak about the multiple accountabilities that NGOs face [5,9,21,64], and the resulting challenges from this “unbalanced accountability” [5], p. 376. Due to this asymmetry amongst players, an NGO tends to respond to the priorities of its donors and the country government over the NGO’s beneficiaries or its own principles [5,23]. However, these previous analyses examined NGO governance (including NGO accountability), partnerships, or donor aid, and did not focus on equity.

That asymmetrical interdependence influences the INGOs’ implementation of equity principles raises a unique contradiction. Inequities arise because of power differentials: “All societies have social hierarchies in which economic and social resources, including power and prestige, are distributed unequally. The unequal distribution of resources affects people’s freedom to lead lives they have reason to value, which in turn has a power effect on health and its distribution in society” [65], p. 1154. Hence, INGOs are working on addressing inequities within an unequal system, where they have less power, and more at stake, than other players. The context of asymmetrical interdependence means there are multiple influences from these players on INGOs’ implementation of equity principles. This creates an implementation gap between the intent of INGOs to ensure equity in their HIV/AIDS work and actual practice.

The Kenyan government and donors’ explicit recognition of equity as a principle in various strategic documents was recognized as a facilitator for the case INGO’s implementation of its equity principles. However, some donors emphasized equity more than others, and the equity strategies outlined by donors and the Kenyan government did not always align with the operationalization of these principles. Because of the interdependence amongst the players in this system, the INGO could not simply focus on its own agenda, but had to take other players into account when planning and implementing its equity principles in its HIV/AIDS work. The INGO faced a number of system influences from donors and the Kenyan government as a result of this asymmetrical interdependence, and aligned its implementation of equity principles accordingly. Other authors have recognized the importance of strategically aligning equity work, particularly with the priorities of the government. The importance of players, including NGOs, aligning their actions on social determinants of health with country governments was identified as a priority strategy in the WHO discussion paper for the World Conference on Social Determinants of Health [66]. Another WHO document on equity and urban health identified aligning with government priorities at a national and local level as a criteria for selecting interventions to address equity [67]. In the present case study, the INGO was heavily reliant on donor funding and thus had to align its work with the donors’ requests for proposals, while needing to maintain its organizational status within Kenya and trying to be true to its equity agenda. Other authors have identified that donors decide on the focus of their funding, often based on their own priorities and needs [54,55], although much of this past work had not focused on equity per se. In the present study, donors’ requests for proposals were based on their priorities, decided in the Northern country, which could change based on the ideology of those holding political power in that country. The short-term nature of funding challenged the INGO’s implementation of its equity principles, given that addressing inequities, and the structural determinants of health, can take a long time. The challenges with short-term donor funding have also been identified in the NGO literature [30,68].

### Privileging of particular vulnerable populations in equity implementation

The case INGO chose to maintain positive relationships with the Kenyan government rather than take a confrontational approach to equity. Hence, the INGO privileged particular vulnerable populations based on its reputation, its history, the priorities of the Kenyan government and the donors, and what was deemed less controversial and in keeping with Kenya’s legal framework. Thus, people living in poverty, people living in geographically remote areas, and gender inequities were the primary foci for the INGO’s implementation of equity principles in its HIV/AIDS work. These populations also benefited from a more upstream structural approach by the INGO, where programming went beyond downstream issues of health care access and behaviour change to include addressing structural determinants of health (e.g. working with cultural custodians to shift cultures around gender; working on income-generating projects). However, other vulnerable populations, including men who have sex with men, sex workers, and injecting drug users, were less privileged by the INGO. The work the INGO did with these populations tended to take an approach that focused on behaviour change and access to health services. A more upstream human rights orientation, including advocating legislative reform to address the structures that were perpetuating inequities, was missing for these populations. Hence, this choice limited the programming and advocacy approaches that the INGO took to address equity. An illustration of the potential influence of a Southern country government on INGOs’ implementation of equity principles is Ethiopia’s legislation that prohibited foreign NGOs from working in areas of human rights, gender equality, and ethnic equality [69,70].

#### Aligning with the system

The INGO focused its implementation of equity principles in its HIV/AIDS work by continuing in areas where it had a historical reputation as well as by working within the realm of what was supported by the donors and the Kenyan government. Many equity tools, global strategies on equity, and key authors working in equity have identified common elements to address inequities that are congruent with the work of the case INGO (e.g. community empowerment and focusing on vulnerable populations) [3,71-83]. However, this previous work did not examine how the system within which INGOs work, including the context of asymmetrical interdependence, may influence their focus on particular elements of equity principles in their HIV/AIDS work.

The case INGO had a positive historical reputation in working with people living in remote areas and people living in poverty. This aligned with the system, since donors and the Kenyan government had supported the INGO’s work in these areas – areas where the INGO had worked since its inception. Other literature supports this, arguing that NGOs are strongly influenced by the discourse in place at the time of their formation [84]. Rather than implement its agenda independently, the INGO ensured its congruence with the priorities of the other key players, implementing its equity principles while mitigating risks.

Previous research on NGOs’ implementation of gender mainstreaming has found that donors have driven the gender mainstreaming agenda [28-31]. In the present research, donors also played a key role in putting gender on the agenda. However, while previous research has also identified that NGOs had limited ownership of gender mainstreaming and tended to focus on community’s immediate needs versus shifting underlying determinants [29-31], the case INGO did embrace gender mainstreaming in its community interventions, including trying to shift cultural practices and working with chiefs’ *barazas.* However, the INGO data monitoring approaches related to equity simply involved disaggregation by sex, a finding reported by others [29-31]. For accountability purposes and to develop a continuous learning cycle, governments, NGOs and other institutions need to measure inequities in health across various groups to monitor progress in access to health care, health status and health outcomes over time. This would allow an equity assessment of actions taken, to ensure that inequities are not worsening as a result of interventions, and to make adjustments based on results [1,3,73,74,76-78,81-83,85-88].

## Conclusion

Global calls to address inequities and health argue that action on inequities is not limited to governments, but includes multiple players, including civil society and the global community [3,4, 89]. Given this commitment to reducing inequities, it is important to look at the role that various organizations play, including INGOs, to continue to find the best ways for INGOs to address inequities, and to examine and address any challenges that these organizations face when trying to implement equity principles.

The present research shows that a gap exists between the intent of INGOs’ implementation of equity principles and actual practice as a result of multiple influences from different players, namely donors and country governments. Given reliance on donor funding and the need for permission from the government to work in-country, the INGO aligned the implementation of its equity principles in its HIV/AIDS initiatives by working within a system characterized by asymmetrical interdependence. The INGO privileged certain vulnerable populations over others, worked in areas where it had a positive and longstanding reputation, and also worked in areas supported by the donors and the Kenyan government.

This research study adds to the literature by examining the tripartite relationship amongst INGOs, Southern country governments, and donors, which adds depth to understanding the context within which INGOs are implementing their equity principles. This research can help INGOs, Southern country governments, and donors to better understand that implementation of equity has to consider the system within which the INGO works, and the players that influence it. The elucidation of these various influences may assist these players when they are contemplating partnerships on equity issues in HIV/AIDS or other areas by showing the influences, whether intended or not, on the INGO’s implementation of equity principles. Future research could extend our understanding of these influences by examining NGOs with different mandates and by investigating the impact of global structures on the equity agenda.

## Endnotes

^a^As a result, we use the term “NGO” to represent non-governmental organizations more broadly. We only use the term INGO when the literature specifies INGO, or when speaking directly about the findings of the case INGO.

^b^This article uses the term Southern country to denote developing countries, and Northern country to describe developed countries.

^c^Chief’s *barazas* are community meetings with chiefs and assistant chiefs of communities.

^d^We use the term “most-at-risk population” as this was the term used during data collection, and the term used in various Kenyan documents (e.g. INGO, government). However, UNAIDS released updated terminology that encourages the use of the term “key populations” instead of “most-at-risk populations”, to discourage stigmatization [19], p. 10.

## Abbreviations

AIDS: Acquired immune deficiency syndrome or acquired immunodeficiency syndrome
ART: Anti-retroviral therapy
HIV: Human immunodeficiency virus
IDU: Injecting drug users
INGO: International Non-Governmental Organization
KEMRI: Kenyan Medical Research Institute
KNASP: Kenyan National AIDS Strategic Plan
MARPs: Most-at-risk populations
MSM: Men who have sex with men
PEPFAR: US President's Emergency Plan for AIDS Relief

## References

[1] Whitehead M (1990). The Concepts and Principles of Equity and Health.

[2] Commission on Social Determinants of Health (2007). Achieving Health Equity: From Root Causes To Fair Outcomes.

[3] Commission on Social Determinants of Health (2008). Closing the Gap in a Generation.

[4] World Health Organization (2011). Rio Political Declaration on Social Determinants of Health.

[5] Lewis D, Kirkpatrick C, Clarke R, Polidano C (2002). The rise of non-governmental organizations: issues in development management. Handbook on Development Policy and Management.

[6] Ronalds P (2010). The Change Imperative: Creating the Next Generation NGO.

[7] Lewis D, Kanji N (2009). Non-Governmental Organizations and Development.

[8] Bhan A, Singh JA, Upshur RE, Singer PA, Daar AS: **Grand challenges in global health: engaging civil society organizations in biomedical research in developing countries.** *PLoS Med* 2007, **4:**e272. 10.1371/journal.pmed.0040272 PMC1989739 17850177

[9] Lindenberg M, Bryant C (2001). Going Global: Transforming Relief and Development NGOs.

[10] Dibie RA, Dibie RA (2007). Introduction: NGOs and Human Development in Africa: Theory and Model for Collaboration. Non-Governmental Organizations (NGOs) and Sustainable Development in Sub-Saharan Africa.

[11] Benotsch EG, Stevenson LY, Sitzler CA, Kelly JA, Makhaye G, Mathey ED, Somlai AM, Brown KD, Amirkhanian Y, Fernandez MI, Opgenorth KM (2004). HIV prevention in Africa: programs and populations served by non-governmental organizations. J Community Health.

[12] Kelly JA, Somlai AM, Benotsch EG, Amirkhanian YA, Fernandez MI, Stevenson LY, Sitzler CA, McAuliffe TL, Brown KD, Opgenorth KM (2006). Programmes, resources, and needs of HIV-prevention nongovernmental organizations (NGOs) in Africa, Central/Eastern Europe and Central Asia, Latin America and the Caribbean. AIDS Care.

[13] Pfeiffer J (2004). Civil Society, NGOs, and the Holy Spirit in Mozambique. Hum Organ.

[14] Union of International Associations (2011). Yearbook of International Organizations.

[15] United Nations Population Fund (2009). Financial Resource Flows for Population Activities in 2009.

[16] United Nations: *UN Millennium Development Goals.* [www.un.org/millenniumgoals/]

[17] UNAIDS (2012). UNAIDS World AIDS Day Report 2012.

[18] UNAIDS (2010). Global Report: UNAIDS Report on the Global AIDS Epidemic.

[19] UNAIDS (2011). UNAIDS Terminology Guidelines.

[20] Kindornay S, Ron J, Carpenter C (2012). Rights-based approaches to development: implications for NGOs. Hum Rights Q.

[21] Smith SC (2004). Governance of Nongovernmental Organizations: A Framework and Application to Poverty Programs in East Africa. Conference on Corporate Governance and Accountability in Sub-Saharan Africa: The Africa Project of the Institute for International Corporate Governance and Accountability.

[22] Homen H (2010). Snakes in Paradise: NGOs and the Aid Industry in Africa.

[23] Najam A (1996). NGO accountability: a conceptual framework. Dev Pol Rev.

[24] Edwards M, Hulme D (1996). Too close for comfort? The impact of official aid on nongovernmental organizations. World Dev.

[25] Pinkney R (2009). NGOs, Africa, and the Global Order.

[26] Fowler A (2000). NGDOs as a moment in history: beyond aid to society entrepreneurship or civic innovation?. Third World Q.

[27] Malhotra K (2000). NGOs without aid: beyond the global soup kitchen. Third World Q.

[28] Tiessen R (2004). Re-inventing the gendered organization: staff attitudes towards women and gender mainstreaming in NGOs in Malawi. Gen Work Organ.

[29] Tiessen R (2005). Mainstreaming gender in HIV/AIDS programs: ongoing challenges and new opportunities in Malawi. J Int Womens Stud.

[30] Wendoh S, Wallace T (2005). Re-thinking gender mainstreaming in African NGOs and communities. Gen Dev.

[31] Desai V (2005). NGOs, gender mainstreaming, and urban poor communities in Mumbai. Gen Dev.

[32] Kenya National Bureau of Statistics (2010). Kenya Demographic and Health Survey.

[33] National AIDS, Control Council UNAIDS (2009). Global HIV/AIDS Program (GHAP) Global AIDS M&E Team (GAMET): Kenya: HIV Prevention Response and Modes of Transmission Analysis.

[34] NACC, NASCOP (2012). Kenya AIDS Epidemic Update 2011.

[35] NGOs Coordination Board (2009). Report on the National Validation Survey of NGOs.

[36] Thomas G (2011). How to do Your Case Study.

[37] Stake RE (1995). The Art of Case Study Research.

[38] Creswell JW (2007). Qualitative Inquiry and Research Design: Choosing Among Five Approaches.

[39] Yin RK (2003). Case Study Research: Design and Methods.

[40] Adler P, Adler P (1987). Membership Roles in Field Research.

[41] Miles MB, Huberman AM (1994). An Expanded Sourcebook: Qualitative Data Analysis.

[42] Canadian Institutes of Health Research, Natural Sciences and Engineering Research Council of Canada, Social Sciences, Humanities Research Council of Canada (1998). Tri-Council Policy Statement: Ethical Conduct for Research Involving Humans. (Public Works and Government Services Canada.

[43] *Ethics Guidelines.* [www.research.uottawa.ca/ethics/guidelines.html]

[44] Auerbach CF, Silverstein LB (2003). Qualitative Data: An Introduction to Coding and Analysis.

[45] *NVivo 9.* [http://www.qsrinternational.com/]

[46] Saldana J (2009). The Coding Manual for Qualitative Researchers.

[47] Government of Kenya (2005). Reversing the Trends - The Second National Health Sector Strategic Plan of Kenya (NHSSP II) 2005–2010.

[48] National AIDS Control Council (2009). Kenya National AIDS Strategic Plan III: 2009/10–2012/13 - Delivering on Universal Access to Services.

[49] Ministry of Health (2006). Taking the Kenya Essential Package for Health to the Community - A Strategy for the Delivery of Level One Services.

[50] Paris Declaration on Aid Effectiveness (2005). Paris Declaration on Aid Effectiveness: Joint Progress Toward Enhanced Aid Effectiveness High Level Forum February 28-March 2, 2005.

[51] UNAIDS (2004). "Three Ones” Key Principles.

[52] Government of Kenya and the Development Partners and Implementing Partners (2007). Kenya Health Sector Wide Approach: Code of Conduct.

[53] Keohane RO (1990). Soviet-American Dialogue in the Social Sciences: Research Workshops on Interdependence Among Nations.

[54] Garrett L (2007). The Challenge of Global Health. Foreign Affairs.

[55] Amutabi MN (2006). The NGO Factor in Africa: The Case of Arrested Development in Kenya.

[56] Kanyinga K, Mitullah W, Njagi S (2007). The Non-Profit Sector in Kenya: What we know and what we don’t know.

[57] Hinton R, Groves L, Leslie G, Rachel H (2004). The Complexity of Inclusive Aid. Inclusive Aid: Changing Power and Relationships in International Development.

[58] Lister S (2000). Power in partnership? An analysis of an NGO’s relationships with its partners. J Int Dev.

[59] Parkin F (1982). Max Weber.

[60] Reed A, Reed D (2009). Partnerships for development: four models of business involvement. J Bus Ethics.

[61] Olsen ME (1991). Societal Dynamics: Exploring Macrosociology.

[62] Morvaridi B (2008). Social Justice and Development.

[63] Mosse D, Lewis D, Lewis D, Mosse D, Mosse D (2006). Theoretical Approaches to Brokerage and Translation in Development. Development brokers and translators: the ethnography of aid and agencies.

[64] Seckinelgin H (2004). Who can help people with HIV/AIDS in Africa? Governance of HIV/AIDS and Civil Society. Voluntas.

[65] Marmot M (2007). Achieving health equity: from root causes to fair outcomes. Lancet.

[66] World Health Organization (2011). Closing the Gap: Policy into Practice on Social Determinants of Health: Discussion Paper.

[67] World Health Organization (2010). Urban Heart: Urban Health Equity Assessment and Response Tool.

[68] Easterly W (2002). The cartel of good intentions: the problem of bureaucracy in foreign aid. Policy Reform.

[69] Ethiopia: **New law on charities passed despite objections.** January 6 [http://allafrica.com/stories/200901070659.html]

[70] Tiwana MS (2008). Analysis of the Ethiopia Charities and Societies Proclamation 00/ 2008.

[71] Krieger N (2001). A glossary for social epidemiology. J Epidemiol Community Health.

[72] Whitehead M, Dahlgren G (2006). Levelling up (part 1): A Discussion Paper on Concepts and Principles for Tackling Social Inequities in Health.

[73] Tamburlini G (2004). Promoting equity in health. Health Policy Dev.

[74] Braveman P (2006). Health disparities and health equity: concepts and measurement. Annu Rev Public Health.

[75] Venkatapuram S, Bell R, Marmot M (2010). The right to sutures: social epidemiology, human rights, and social justice. Health Hum Rights.

[76] World Health Organization (2011). Improving Equity in Health by Addressing Social Determinants.

[77] Whitehead M (1992). The concepts and principles of equity and health. Int J Health Serv.

[78] Canadian Institute for Health Information (2012). Urban Physical Environments and Health Inequalities: A Scoping Review of Interventions.

[79] Jones H (2009). Equity in Development: Why it is Important and How to Achieve It.

[80] Rifkin SB (2003). A framework linking community empowerment and health equity: it is a matter of CHOICE. J Health Popul Nutr.

[81] USAID, Maternal and Child Health Integrated Program (2011). Considerations for Incorporating Health Equity into Project Designs: A Guide for Community-Oriented Maternal, Neonatal, and Child Health Projects.

[82] Friel S, Bell R, Houweling T, Marmot M (2009). Calling all Don Quixotes and Sancho Panzas: achieving the dream of global health equity through practical action on the social determinants of health. Glob Health Promot.

[83] The Global Equity Gauge Alliance (GEGA) (2003). The Equity Gauge: Concepts, Principles, and Guidelines.

[84] Ebrahim A (2001). NGO behavior and development discourse: class from Western India. Voluntas: Int J Volunt Nonprofit Org.

[85] Bierman A, Johns A, Hyndman B, Mitchell C, Degani N, Shack AR, Creatore MI, Lofters A, Urquia ML, Ahmad F, Khanlou N, Parlette V, Bierman AS (2012). Chapter 12: Social Determinants of Health and Populations at Risk. Ontario Women’s Health Equity Report.

[86] NHS Department of Health (2004). Health Equity Audit: A Self-Assessment Tool.

[87] Signal L, Martin J, Cram F, Robson B (2008). The Health Equity Assessment Tool: A User’s Guide.

[88] UNICEF (2011). Evaluation for Equitable Development Results.

[89] Marmot M, Allen J, Bell R, Goldblatt P (2012). Building of the global movement for health equity: from Santiago to Rio and beyond. Lancet.

